# Iodine in the Treatment of Uterine Leucorrhœa

**Published:** 1866-09

**Authors:** 


### Iodine in the Treatment of Uterine Leucorrhœa.

The treatment of leucorrhœa is a constant subject of difficulty and vexation to the medical practitioner. Although the use of various astringents will often effect improvement, yet this is seldom lasting, and the recurrence of the symptoms is a continual source of annoyance. We have lately observed a plan which is being pursued by Dr. Murray at the Great Northern Hospital, and which promises to be a very useful addition to our means of treatment in this very troublesome condition. Dr. Murray first ascertains, by means of the speculum, that the discharge proceeds from within the uterus. He then introduces a small, short-haired brush (much like that used for washing phials) by a screw-like motion, so that the thick phlegm-like layer on the uterine wall is awept off with every turn of the brush. When this reaches the fundus he steadily withdraws it, charged as it is with the mucous deposit. Its place is then taken by a gum-elastic catheter with several apertures, through which is injected a lotion consisting of one part of the compound tincture of iodine to two parts of water. The uterine wall is thoroughly washed with this. The muscular contraction which follows this injection is remarkable, the tube being tightly grasped, so that its re-introduction at the time is extremely difficult. Dr. Murray has reason, after an experience of many cases treated by this plan, to feel highly satisfied with its success.

In this connection the use of iodized cotton, suggested by Dr. Robert Greenhalgh, as an application to the cervix uteri in chronic inflammatory enlargements and thickenings, and in subinvolution, with or without congestion or induration of tissue, is of interest. It is prepared as follows: Two ounces of iodide of potassium and one ounce of iodine are dissolved in eight ounces of glycerine, in which solution eight ounces of cotton wool are thoroughly saturated and then carefully dried. It should be applied through a speculum directly to the cervix uteri, using the precaution of securing it properly by a silk thread, and should be kept in position in the vagina for from twenty-four to forty-eight hours. Dr. Greenhalgh claims for it the following advantages: It is light, clean and portable; produces no irritation; destroys all fœtor; is considerably stronger than the compound tincture of iodine; is more readily absorbed, and can be kept for a longer time in contact with the diseased tissues; and, moreover, it does not soil the linen like many of the suppositories and medicated appliances in use for uterine affections.—*Lancet, Jan.* 6, 1866.

